# Sensorless Control of the Permanent Magnet Synchronous Motor

**DOI:** 10.3390/s19163546

**Published:** 2019-08-14

**Authors:** Konrad Urbanski, Dariusz Janiszewski

**Affiliations:** Institute of Control, Robotics and Information Engineering, Poznan University of Technology, PL60965 Poznan, Poland

**Keywords:** PMSM, permanent magnet motors, sensorless control, observers, variable-speed drives, Kalman filter

## Abstract

This paper describes the study and experimental verification of sensorless control of permanent magnet synchronous motors with a high precision drive using two novel estimation methods. All the studies of the modified Luenberger observer, reference model, and unscented Kalman filter are presented with algorithm details. The main part determines trials with a full range of reference speeds with a special near-zero speed area taken into account. In order to compare the estimation performances of the observers, both are designed for the same motor and control system and run in the same environment. The experimental results indicate that the presented methods are capable of tracking the actual values of speed and motor position with small deviation, sufficient for precise control.

## 1. Introduction

Permanent magnet synchronous motors (PMSM) are commonly used in machining tools and industrial power systems. PMSM-based drives are used in industry both in the drives with the highest precision and those without. The basic advantages of the PMSM drive are high dynamics and high energy efficiency. These attributes result from a small moment of inertia, a high torque to weight ratio, and negligible electrical losses in the rotor. The disadvantage of the traditional PMSM drive is the need to know the flux position. Information about the current position of the shaft flux is used to control the transformation between the coordinate systems and to determine the rotational speed. Eliminating the mechanical sensor that is used to measure the speed and position of the shaft will make it possible to obtain low-cost solutions (the cost of the sensor and its installation) and more compact construction (two-side shaft) and will increase the reliability of the drive [1].

Controlled electric drives without mechanical speed and position sensors at the motor shaft are attractive because of low cost and high reliability. The adjective “sensorless” refers to the lack of any mechanical sensors, but the presence of electrical transducers. Readers can consult [2,3,4,5,6,7,8,9,10,11,12,13,14,15,16], current works on several developed methods, most of which are based on observer theory. One such work, considered here, is based on back electromotive force (EMF) estimation [17,18,19]. Another is the widely-known Kalman filter theory [20]. During all investigations, only terminal signals were used: stator voltages and stator currents, both for simulation and laboratory experiment proof.

In order to control the drive systems for the non-zero speed range, different methods for estimating the shaft position are used [21,22,23,24,25,26,27], including the Luenberger observer [17], the modified Luenberger observer (with multiple integrator) [24], and the above-mentioned Kalman filter [25]. In the case of sensorless drives in the low-speed range (assuming the following distinction for sensorless operation: low speed, the speed of a single revolution per second; very low speed, the speed of a single revolution per minute), it is usually assumed that the methods based on estimating the electromotive forces do not work properly [22,26,27]. This is explained by the small amplitude values of back EMF and a relatively high noise level in this speed range, which has an impact on the accuracy and stability of the back EMF estimator. Experimental tests have shown in [18] that even at the low speed, shaft position can be estimated using the back EMF observer with satisfactory accuracy. The question of the (estimated) speed calculation using common methods gives sufficient accuracy, for example the method based on the length of the back EMF vector or the derivative of the position of the shaft, whereas this range of speed usually does not give good results. The solution may be a new control structure, as presented in this paper.

This article is organized as follows. First, the design considerations are presented, and some problems in sensorless control are indicated. Next, the model of the PMSM oriented into observers’ structures is proposed. Then, the theoretical background of the *unscented Kalman filter* (UKF) and *Luenberger observer* with novel correction models is described. Finally, the effectiveness of the proposed sensorless control system is verified in the laboratory.

## 2. Sensorless Control

Achieving comparable speed control of an electrical machine with and without external shaft sensors is possible. Sensorless control is based on rejecting any mechanical sensors placed on the machine shaft during mechanical quantities’ control. As described in [2,28,29], that type of rejection provides advantages such as: increased reliability of the drive, ability to work in adverse environmental conditions, and a decrease in the cost of the drive system.

**Figure 1:**
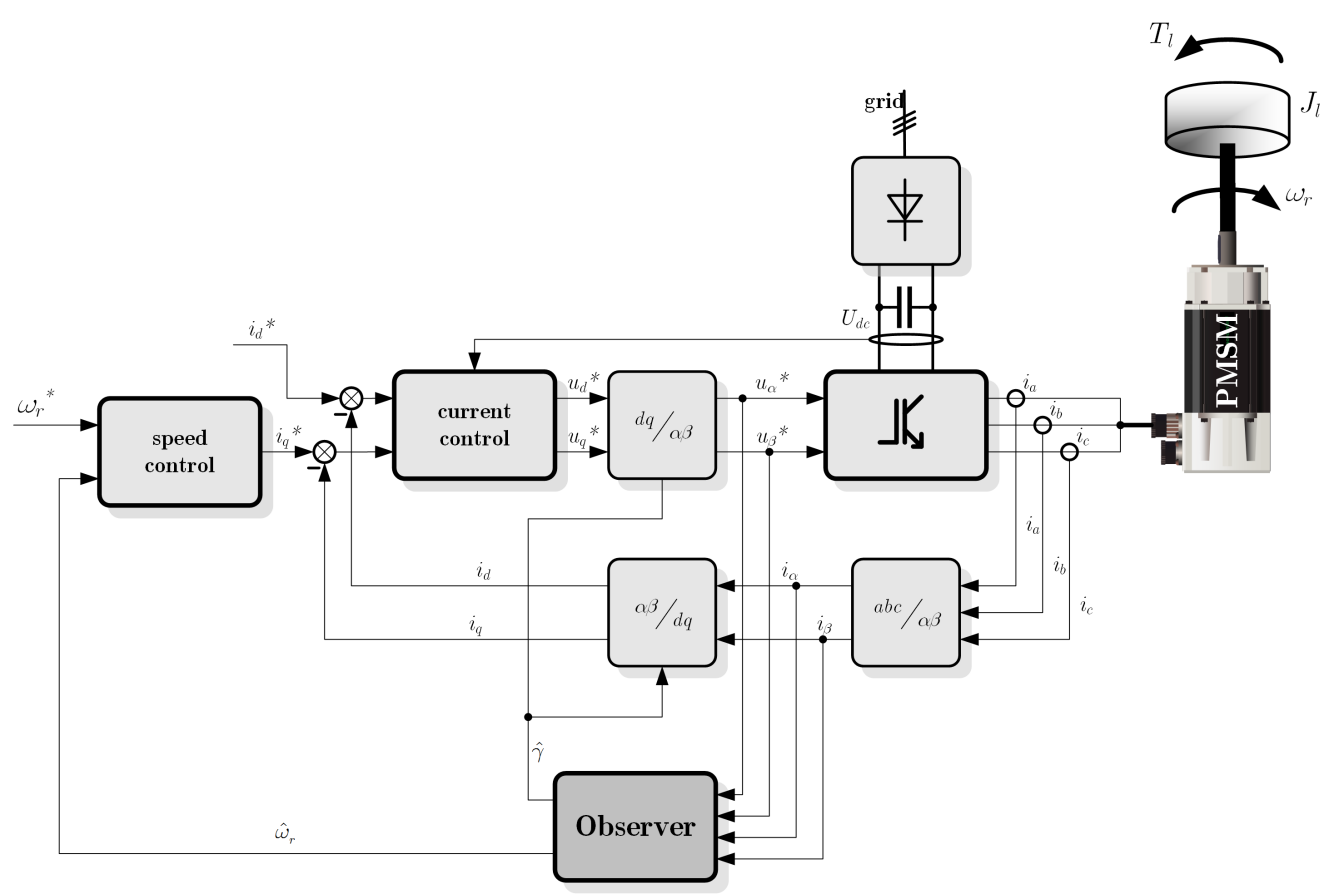
The proposed sensorless drive scheme.

*Field-oriented control* can be called the classical method of PMSM control, where field orientation determines the space vectors of magnetic flux, current, and voltage [2,30,31,32]. This simple control scheme is presented in Figure 1. There is a two-axis flat space, perpendicular dq axes, where *d* is rotor flux coordinated. Thus, its magnitude position can be obtained directly from the rotor shaft by measuring the rotor angle. An optimally-efficient operation is achieved by the stator’s current control, which ensures that it contains only a quadrature *q* axis component [28,30]. It is possible to set up the coordinate system to decompose the vectors into electromagnetic field generation and torque production [33]. *Field-oriented control* ensures the high dynamic performance of the electric drive with PMSM and the PWM inverter.

### 2.1. Estimation Vector

In analyzing real motor and model behaviors, some variables should be specifically considered. From the observer’s point of view, some variables should be estimated because they are an important part of the state space model. Based on the analysis, complex mechanical structures of supplied electric drives are noted; such generalized load torque $T_{l}$ can be helpful during control. The problem of this extended observation has often been noted in the literature [13,34,35]. The state space vector of the proposed observer is:

(1) $$\hat{\underline{x}}={\left[\begin{array}{ccccc} i_{d} & i_{q} & \omega_{r} & \gamma & T_{l} \end{array}\right]}^{T}\;,$$

where $i_{d}$ and $i_{q}$ are the dq axis currents, $\omega_{r}$ is the rotor speed, γ is the mechanical shaft (excitation flux magnitude) position, and $T_{l}$ is the generalized load torque.

### 2.2. Mathematical Model of PMSM

The classical *control system* statistical approach determines the state transition and measurement probability model of the system with densities:

(2) $$\begin{array}{c} p\left({\underline{x}}_{t}|{\underline{x}}_{t-1},{\underline{u}}_{t}\right) \end{array}$$

(3) $$\begin{array}{c} p\left({\underline{y}}_{t}|{\underline{x}}_{t}\right) \end{array}$$

where ${\underline{x}}_{t}\in R^{n_{x}}$ denotes the states (hidden variables and/or parameters) of the system in time *t* and ${\underline{y}}_{t}\in R^{n_{y}}$ known observations. The states follow a first order Markov chain process, and the observations are assumed to be independent given the states, so the model can be expressed as:

(4) $${\underline{x}}_{t}=f\left({\underline{x}}_{t-1},{\underline{u}}_{t},{\underline{v}}_{t-1}\right)\;,$$

(5) $${\underline{z}}_{t}=h\left({\underline{x}}_{t},{\underline{n}}_{t}\right)\;,$$

where ${\underline{u}}_{t}\in R^{n_{u}}$ denotes the input observations and ${\underline{v}}_{t}\in R^{n_{x}}$ and ${\underline{n}}_{t}\in R^{n_{y}}$ are noises for the process and measurements, respectively. State space representation can be presented as:

(6) $${\underline{x}}_{t}=F_{t}\left({\underline{x}}_{t-1}\right){\underline{x}}_{t-1}+B_{t}\left({\underline{x}}_{t-1}\right){\underline{u}}_{t}+{\underline{v}}_{t-1}\;,$$

(7) $${\underline{z}}_{t}=H_{t}\left({\underline{x}}_{t}\right){\underline{x}}_{t}+{\underline{n}}_{t}\;,$$

The mathematical model of the considered PMSM can be divided into three main parts: the stator’s electrical circuit, electromechanical torque production, and the mechanical subsystem [36]. Some simplified assumptions should be introduced: saturation is neglected; inducted electromagnetic force is sinusoidal; eddy currents and hysteresis losses are neglected; no dynamical dependencies in the air-gap; no rotor cage. The rotor-oriented dq electrical network equations of PMSM can be described as:

(8) $$u_{d}=R_{s}i_{d}+L_{d}\frac{di_{d}}{dt}-p\omega_{r}L_{q}i_{q}\;,$$

(9) $$u_{q}=R_{s}i_{q}+L_{q}\frac{di_{q}}{dt}+p\omega_{r}L_{d}i_{d}+p\omega_{r}\Psi_{m}\;.$$

where $u_{d}$, $u_{q}$ are the dq axis voltages, $i_{d}$, $i_{q}$ are the dq axis currents, $L_{d}$, $L_{q}$ are the dq axis inductances, $R_{s}$ is the stator resistance, and $\Psi_{m}$ is magnetic flux produced by permanent magnets placed on the rotor.

The produced electromagnetic torque is given by the relationship:

(10) $$T_{e}=\frac{3}{2}\;p\left[\Psi_{m}-\left(L_{q}-L_{d}\right)i_{d}\right]\;i_{q}\;,$$

where *p* is the number of pole pairs, and fraction $\frac{3}{2}$ stems from frame conversion: perpendicular stator αβ into the rotor dq reference frame.

Drive dynamics can be described as:

(11) $$T_{e}-T_{l}=J\frac{d\omega_{r}}{dt},$$

where $T_{l}$ is load torque and *J* is the summary moment of inertia of the kinematic chain.

Based on (10) and (11), the movement equation is:

(12) $$\frac{d\omega_{r}}{dt}=\frac{p}{J}\left[\frac{3}{2}\left(\Psi_{m}-\left(L_{q}-L_{d}\right)i_{d}\right)\;i_{q}\right]-\frac{T_{e}}{J}\;.$$

Rotor position γ can be described by the derivative equation of the rotational speed:

(13) $$\frac{d\gamma }{dt}=p\;\omega_{r}\;.$$

For the presented approach, the assumption that load torque $T_{l}$ is invariable in a small interval is true:

(14) $$\frac{d}{dt}T_{l}\approx 0\;.$$

The model can be described as the classical discrete function model, with sample time $T_{s}$ as the state space model. The system parameters’ matrix $F_{t}$ is:

(15) $$F_{t}\left({\hat{\underline{x}}}_{t}\right)=\left[\begin{array}{ccccc} 1-T_{s}\;\frac{R_{s}}{L_{d}} & T_{s}\;\omega_{r}\frac{L_{q}}{L_{d}} & 0 & 0 & 0\\ -T_{s}\;\omega_{r}\frac{L_{d}}{L_{q}} & 1-T_{s}\;\frac{R_{s}}{L_{q}} & -T_{s}\;\frac{\Psi_{m}}{L_{q}} & 0 & 0\\ 0 & T_{1} & 0 & 0 & -T_{s}\;\frac{1}{J}\\ 0 & 0 & T_{s} & 1 & 0\\ 0 & 0 & 0 & 0 & 1 \end{array}\right]\;,$$

where:

$$T_{1}=T_{s}\;\frac{3}{2}\frac{p}{J}\left[\Psi_{f}-\left(L_{q}-L_{d}\right)i_{d}\right]$$

The output matrix $H_{t}$ is a Clarke/Park transformation:

(16) $$H_{t}\left({\hat{\underline{x}}}_{t}\right)=\left[\begin{array}{cccc} \cos\gamma & -\sin\gamma & 0 & 0\\ \sin\gamma & \cos\gamma & 0 & 0 \end{array}\right]\;,$$

and matrix $B_{t}$:

(17) $$B_{t}\left({\hat{\underline{x}}}_{t}\right)=\left[\begin{array}{cc} T_{s}\;\frac{1}{L_{d}}\cos\gamma & T_{s}\;\frac{1}{L_{d}}\sin\gamma \\ -T_{s}\;\frac{1}{L_{q}}\sin\gamma & T_{s}\;\frac{1}{L_{q}}\cos\gamma \\ 0 & 0\\ 0 & 0\\ 0 & 0 \end{array}\right]\;.$$

## 3. The Kalman Filter Sensorless Control

In control systems and statistics, the Kalman filter is a recursive algorithm with infinite impulse response [20]. This means that the derivative of the known estimate $\frac{d}{dt}\underline{x}$, called the system trend, and the set of output measurements ${\underline{y}}_{k}$ are sufficient to estimate the current state of the system ${\underline{x}}_{k}$ at each time t∈(0,∞). For the Kalman filter, it is not necessary to know the history of observations. Mathematically, the Kalman filter states can be described by two variables: $\frac{d}{dt}\underline{x}$, a trend that is the derivative of the current state estimate obtained based on knowledge of observation and $P_{k|k}$ the uncertainty of that, the error covariance matrix of the estimation process.

The Kalman filter estimation process is divided into two subsequent stages: *prediction* and *correction*. The entire process filter’s computation is performed recursively to obtain the optimum value of the corrector $K_{k}$ with an assumed error that is as small as possible ε. Prediction performs the state estimate $\hat{\underline{x}}$ based on the trend and input signal $\underline{u}$. Correction leads to improvements in the new estimate of the exact value $\hat{\underline{x}}$ based on the measured output ${\underline{y}}_{k}$ and the value of the corrector $K_{k}$. Prediction is also called time actualization and is based on knowing the derivative of the state $\frac{d}{dt}\hat{\underline{x}}$. It is described as a relation in the presence of input $\underline{u}$:

(18) $$\frac{d}{dt}\hat{\underline{x}}=F_{k}\hat{\underline{x}}+B_{k}\underline{u}\;.$$

Innovation and some form of filtering at this stage cause updates with a vector of state variables $\underline{x}$ of the system covariance P, based on state function (18):

(19) $$P_{k|k-1}=F_{k}P_{k-1|k-1}F_{k}^{T}+Q\;.$$

Correction, which is the actualization of the measurement, involves the introduction of the correction signal based on the measured output ${\underline{z}}_{k}$. It is defined as the difference in response values of the observer and the measured output signal, like a residual:

(20) $${\tilde{\underline{y}}}_{k}={\underline{z}}_{k}-H_{k}{\hat{\underline{x}}}_{k|k-1}\;.$$

Additionally, the innovation covariance system is introduced, based on knowledge of the measurement covariance R:

(21) $$S_{k}=H_{k}P_{k|k-1}H_{k}^{T}+R\;.$$

Based on the above, the mathematical form of the corrector can be determined by:

(22) $$K_{k}=P_{k|k-1}H_{k}^{T}S_{k}^{-1}\;,$$

which together with the calculated residual (20) corrects the state vector:

(23) $${\hat{\underline{x}}}_{k}={\hat{\underline{x}}}_{k-1}+K_{k}{\tilde{\underline{y}}}_{k}\;.$$

With this correction of the state vector ${\hat{\underline{x}}}_{k}$, the covariance is also corrected:

(24) $$P_{k|k}=\left(I-K_{k}H_{k}\right)P_{k|k-1}\;.$$

The above formula for the Kalman filter at optimal Kalman gain $K_{k}$ is valid only for linear systems. Based on the above assumptions, $P_{k|k}$ and the Kalman gain $K_{k}$ are constant and can be derived once based on $F_{k}$, $B_{k}$, $H_{k}$. The calculation is usually carried out iteratively until there is a certain convergence to a consistent result.

There are many developments of the above technical procedure to gain $K_{k}$, which very often reduces the computational complexity. These are: *extended Kalman filters* (EKF) and the *unscented Kalman filters*, both extended for non-linear systems, as described below.

### Unscented Kalman Filter

A main noticeable problem during the estimation of non-linear systems’ behavior exists: it is difficult to determine the probability distribution and the non-linear function of the state and output [37,38,39,40]. This means that the non-linear transformation of the deviation and the *Jacobians* needed for an *extended Kalman filter* do not determine the real covariances. An EKF is based on the classical Kalman filter with the following main assumption: linearity of the object. It is in this way that the covariances are calculated [20,38]. For the class of non-linear objects, the covariances should be associated with the process, but cannot be coupled to setpoint the linearized models of the object. Based on the particular analysis of non-linear systems, the covariance of the state should not be associated with a linearized system and can even be far from them.

An *unscented Kalman filter* is an improvement over the *extended Kalman filter* algorithm. *Simon Julier* and *Julie Uhlman* demonstrated in [39] a new context for the estimation of the theoretical problem based on *unscented transformations*. This method is for calculating the statistics of a random variable, so it is easier to approximate the Gaussian distribution associated with each proposed state vector variable, rather than approximate the non-linear function of the described system behavior. It is possible to simplify the algorithm by eliminating Jacobian calculations. The used approach is based on two assumptions: the first is the determination of the non-linear transformation of the function at work that does not apply to the whole range of the probability density distribution function. The second point concerns the search for work in which this density corresponds to the actual decomposition of the non-linear system. This filter is based on two cycles of procedures: *prediction* and *correction*, like its predecessor.

*Prediction* can be used independently of the UKF update, in combination with the proposed classical linear update. As assumed in the classical Kalman filter approach [20] and as is the case for *extended Kalman filter* prediction, one proceeds in a similar way for each solution. In this case, however, the estimation state vector of the value of disturbances is extended. Such a procedure makes it possible to estimate the state vector and its distributed environment. Strictly speaking, this surrounding will transform non-linear disturbances [39].

A new ${\underline{x}}_{k-1|k-1}^{a}$ vector is defined:

(25) $${\underline{x}}_{k-1|k-1}^{a}={\left[{\hat{\underline{x}}}_{k-1|k-1}^{T}\;E\langle {\underline{w}}_{k}^{T}\rangle \;E\langle {\underline{v}}_{k}^{T}\rangle \;\right]}^{T}\;.$$

Thus, it is natural to define its covariances, which are formed by taking the covariances of the state vector $P_{k-1|k-1}$, the known process noise covariance $Q_{k}$, and the distortion measurement $R_{k}$. It therefore assumes the form:

(26) $$P_{k-1|k-1}^{a}=\left[\begin{array}{ccc} P_{k-1|k-1} & 0 & 0\\ 0 & Q_{k} & 0\\ 0 & 0 & R_{k} \end{array}\right]\;.$$

A set of 2L+1 sigma points, $\chi_{k-1|k-1}$, is derived from the augmented state and covariance, where *L* is the dimension of the augmented state:

(27) $$\begin{array}{ccc} \chi_{k-1|k-1}^{0} & = & {\underline{x}}_{k-1|k-1}^{a}\;, \end{array}$$

(28) $$\begin{array}{ccc} \chi_{k-1|k-1}^{i} & = & {\underline{x}}_{k-1|k-1}^{a}+{\left(\sqrt{\left(L+\lambda \right)P_{k-1|k-1}^{a}}\right)}_{i},\;\\ \mathrm{for}\;i & = & 1..L\;, \end{array}$$

(29) $$\begin{array}{ccc} \chi_{k-1|k-1}^{i} & = & {\underline{x}}_{k-1|k-1}^{a}-{\left(\sqrt{\left(L+\lambda \right)P_{k-1|k-1}^{a}}\right)}_{i-L},\;\\ \mathrm{for}\;i & = & L+1,\cdots 2L\;, \end{array}$$

The matrix square root $\left(\sqrt{nP_{k}}\right)$ should be calculated using numerically-efficient and stable methods, such as the Cholesky decomposition. The sigma points $\chi_{k|k-1}^{i}$ are propagated through the state space transition function (18):

(30) $$\chi_{k|k-1}^{i}=F_{k}\chi_{k-1|k-1}^{i}+B_{k}{\underline{u}}_{k},\;i=0..2L\;.$$

The weighted sigma points $\chi_{k|k-1}^{i}$ are recombined to produce the predicted state ${\hat{\underline{x}}}_{k|k-1}$ and covariance $P_{k|k-1}$:

(31) $${\hat{\underline{x}}}_{k|k-1}=\sum_{i=0}^{2L}W_{s}^{i}\chi_{k|k-1}^{i}\;,$$

(32) $$P_{k|k-1}=\sum_{i=0}^{2L}W_{c}^{i}\;\left[\chi_{k|k-1}^{i}-{\hat{\underline{x}}}_{k|k-1}\right]{\left[\chi_{k|k-1}^{i}-{\hat{\underline{x}}}_{k|k-1}\right]}^{T}\;,$$

where the weights $W_{s}$ and $W_{c}$ for the state and covariance are given by:

(33) $$\begin{array}{ccc} W_{s}^{0} & = & \frac{\lambda }{L+\lambda }\;, \end{array}$$

(34) $$\begin{array}{ccc} W_{c}^{0} & = & \frac{\lambda }{L+\lambda }+\left(1-\alpha^{2}+\beta \right)\;, \end{array}$$

(35) $$\begin{array}{ccc} W_{s}^{i} & = & W_{c}^{i}=\frac{1}{2(L+\lambda )}\;, \end{array}$$

where α, β, and κ are noise distribution parameters and λ is chosen arbitrarily. These are helpful during filter tuning [40]. Typical values for α, β, and κ for the majority of applications in which the disturbance is located in the Gaussian noise assumptions are, respectively, $10^{-3}$, 2, and 0. Any differences from these values can only lead to easier tuning of the filter because they add additional degrees of freedom.

*Correction* is fitted strictly from the classical form. The proposed sigma points $\chi_{k|k-1}^{i}$ are projected through the known observation function $H_{k}$:

(36) $$\Upsilon_{k}^{i}=H_{k}\chi_{k|k-1}^{i}\;,\;i=0..2L\;.$$

Based on weights $W_{s}^{i}$ and $W_{c}^{i}$ from (33) and the observation matrix $\Upsilon_{k}^{i}$, it is possible by recombination to obtain the output signal:

(37) $${\hat{\underline{z}}}_{k}=\sum_{i=0}^{2L}W_{s}^{i}\Upsilon_{k}^{i}\;,$$

and also the output covariance:

(38) $$P_{z_{k}z_{k}}=\sum_{i=0}^{2L}W_{c}^{i}\;\left[\Upsilon_{k}^{i}-{\hat{\underline{z}}}_{k}\right]{\left[\Upsilon_{k}^{i}-{\hat{\underline{z}}}_{k}\right]}^{T}\;.$$

The correction $K_{k}$ depends on the obtained state $P_{k|k-1}$ and the innovation of the system $S_{k}$ covariances and so is similar to (38). Based on the Kalman correction definition:

(39) $$K_{k}=P_{x_{k}z_{k}}P_{z_{k}z_{k}}^{-1}\;,$$

where $P_{x_{k}z_{k}}$ should be:

(40) $$P_{x_{k}z_{k}}=\sum_{i=0}^{2L}W_{c}^{i}\;\left[\chi_{k|k-1}^{i}-{\hat{\underline{x}}}_{k|k-1}\right]{\left[\gamma_{k}^{i}-{\hat{\underline{z}}}_{k}\right]}^{T}\;.$$

As in the classical Kalman filter, the output residual is:

(41) $${\tilde{\underline{y}}}_{k}={\underline{z}}_{k}-h\left({\hat{\underline{x}}}_{k|k-1}\right)\;.$$

The correction of the state is done by:

(42) $${\hat{\underline{x}}}_{k|k}={\hat{\underline{x}}}_{k|k-1}+K_{k}\left({\underline{z}}_{k}-{\hat{\underline{z}}}_{k}\right)\;.$$

The adjusted covariance matrix $P_{k|k}$ is a prediction of $P_{k|k-1}$ corrected by weighted values:

(43) $$P_{k|k}=P_{k|k-1}-K_{k}P_{z_{k}z_{k}}K_{k}^{T}\;.$$

The algorithm is periodic, and the obtained data are shifted for future steps.

The main advantage of the *unscented Kalman filter* is the estimation of the state and output variables with each natural noised environment described by sigma points $\chi_{k|k-1}^{i}$. Each of the variables and their environment are easily described by noise covariance, $Q_{k}$ and $R_{k}$, respectively for states ${\underline{x}}_{k}$ and outputs ${\underline{y}}_{k}$. During the estimation process, sigma points $\chi_{k|k-1}^{i}$ change towards least squares error minimization. Based on that, the operations $Q_{k}$ and $R_{k}$ as part of $P_{k}$ are under modification. Consequently, it is important to choose properly the initial values of $Q_{0}$ and $R_{0}$.

## 4. Control Structure for Low-Speed Range Using the Reference Model

### 4.1. Position Observer

**Figure 2:**
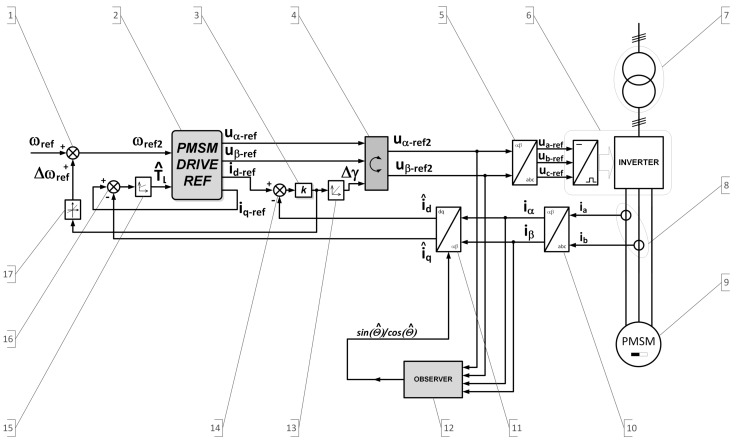
Control structure for the low-speed range.

The choice of a position observer structure is not essential for the present mode of operation of the speed control. The described control structure for the low-speed range, however, includes also the observer (Figure 2, Module 12). The shaft position is determined on the basis of the estimated value of the back EMF. In order to reconstruct the back electromotive forces in a stationary coordinate system αβ associated with the stator, a modified structure of the Luenberger [17,41] observer was used, with the proportional-integral correction function:

(44) $$F\left[\Delta i\right]=K_{p}\left[\Delta i\right]+K_{i}\int \left[\Delta i\right]dt$$

where $K_{p}$ is a proportional gain, $K_{i}$ is an integration gain, and Δi is an estimation error of current.

The used type of observer was thoroughly described in [11] and its potential for low speed range in [18]. The sine and cosine values of the shaft position were determined based on simplified trigonometric dependency:

(45) $$\sin\left(\hat{\gamma }\right)=-\frac{{\hat{e}}_{\alpha }}{|\hat{e}|}\;,$$

(46) $$\cos\left(\hat{\gamma }\right)=\frac{{\hat{e}}_{\beta }}{|\hat{e}|}\;,$$

where ${\hat{e}}_{\alpha }$ and ${\hat{e}}_{\beta }$ are estimated in a stationary coordinate system. The back EMF values and modulus $|\hat{e}|$ were defined as:

(47) $$|\hat{e}|=\sqrt{{\hat{e}}_{\alpha }^{2}+{\hat{e}}_{\beta }^{2}}\;.$$

### 4.2. Modified Control Structure

The proposed sensorless control system was designed to work specifically at the low-speed range, despite the fact that the system was based on an EMF estimation. This system has the ability to start without the use of additional boot algorithm, and could also be used in other speed ranges. The presented structure differs from the known form of control structures, such as MFC (model following control) [42], MRAS (model reference adaptive system) [43], or MRAC (model reference adaptive control) [44]. This system is characterized by the use of the source of reference voltage in the control chain and the use of the correction loops, which are based on currents $i_{d}i_{q}$ in the rotor reference frame.

The scheme of the new sensorless control structure for speed control of the PMSM drive is shown in Figure 2. The structure components are as follows:

1. Adder for the reference speed modifier
2. Reference model of the drive
3. Reference modifying speed gain
4. Rotator
5. Inverse Clarke transformation unit
6. Inverter unit
7. Transformer
8. Current sensors
9. Controlled motor
10. Clarke transformation unit
11. Park transformation unit
12. Position observer
13. Current $i_{d}$ corrector
14. Adder for the correction circuit for current $i_{d}$
15. Load estimator
16. Adder for the load estimator
17. Rate limiter

**Figure 3:**
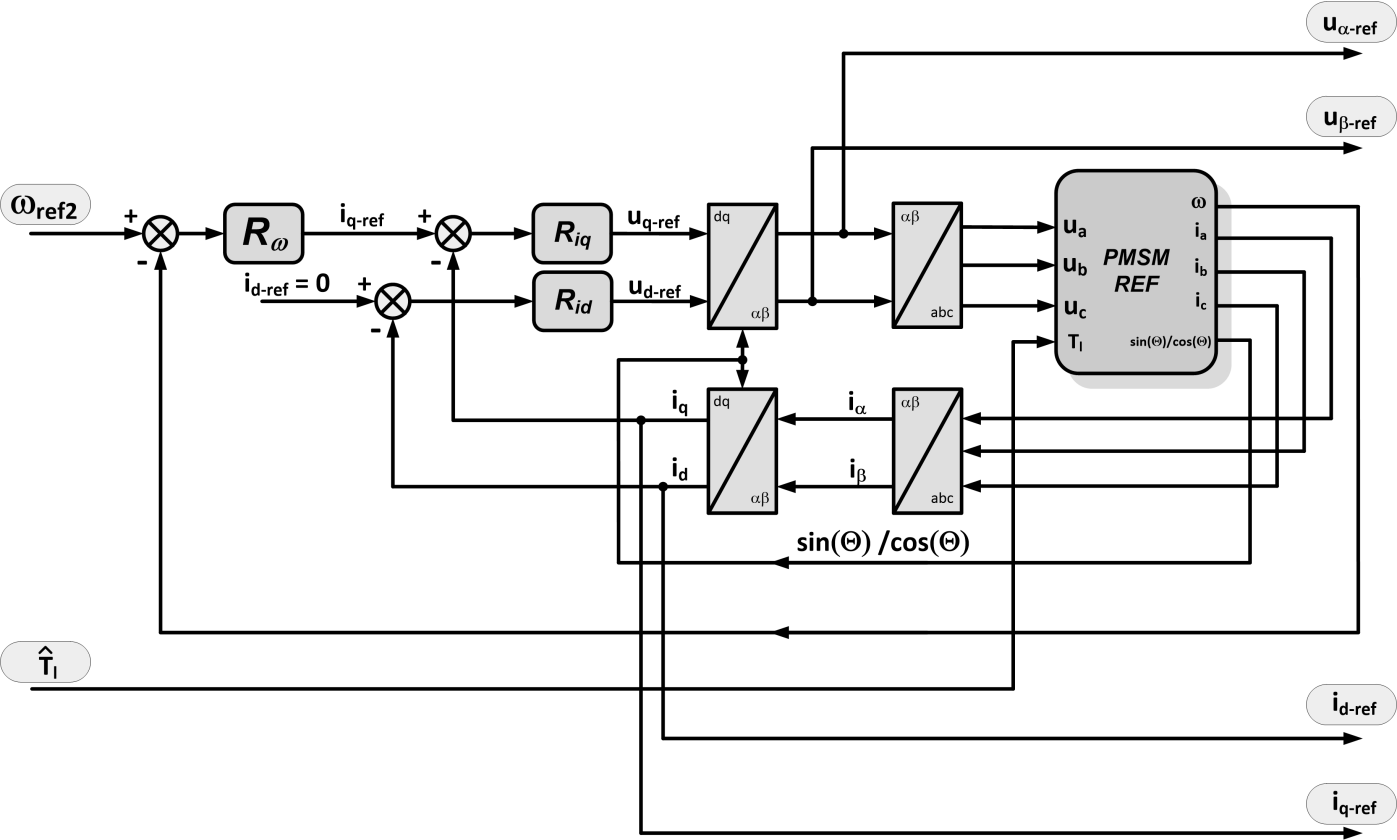
Inner structure of the PMSM drive reference (Figure 2, Module 2) block.

You will notice a dedicated block for the shaft position observer and the block that is the source of reference signals. The main elements of the control scheme are: the source of reference signals (Module 2), the position observer (Module 12), and the auxiliary systems: correction circuit for the current axis *d* (Modules 3, 4, 13, 14), the load estimation (Modules 15, 16), and the correction of reference speed (Modules 1, 3, 14, 17). The source of reference voltages is an appropriately-transformed model of the PMSM drive, which is working in speed control mode (Figure 3).

Its inputs are the current value of the corrected reference speed $\omega_{ref2}$ and estimated load torque ${\hat{T}}_{l}$. Its outputs are: reference voltage $u_{\alpha -ref}$, $u_{\beta -ref}$, set in a stationary αβ coordinate system associated with the stator, and the current reference $i_{d-ref}$ and $i_{q-ref}$ in the dq reference frame rotating synchronously with the rotor. The model of the speed control is determined on the basis of generally-known relationships describing PMSM and the well-known vector control structure with speed and current control. It includes the current control circuits in the dq coordinate system and also the outer speed control loop. The inverter in the reference model is omitted. The shown structure of sensorless control allows controlling the current (in a real machine) in the axes *d* and *q*, as in conventional systems of vector control. The accuracy of modeling the drive with PMSM affects the accuracy of estimating the operating point of the motor in the reference model, which affects the real motor operating point. In the case of the presented structure, the type and structure of the position observer are not important for the operation of the whole system. In the examined drive system, in order to reproduce the position of the shaft, an observer of the electromotive force is used. These values are used to determine the sine and cosine of the shaft position. Electromotive forces are estimated by generally-known dependencies, and also, generally-known dependencies determine the sine and cosine of the estimated value of the angle. The most important feature of the method is the possibility for the stable operation of the drive at low speed, without a shaft position sensor and without calculation or determination of the speed based on the reconstructed value of electromotive force or reconstituted position. Due to correction feedbacks, the system works correctly even when the parameters of the reference model are estimated with limited accuracy.

The sensorless speed control structure was launched in a laboratory setup, with lowered supply voltage through the small transformer (Figure 2, Module 7). The control system operates as follows: the PMSM drive reference model (Figure 2, Module 2) generates reference voltages $u_{\alpha -ref}$, $u_{\beta -ref}$, which depend on the current operating point. If the operating point of the PMSM reference model and the operating point of real PMSM match, the module named the *rotator* (Module 4) leaves the voltage vector unchanged. In the case of non-compliance involving the different values of reference current $i_{d-ref}$ (Module 2) and estimated current ${\hat{i}}_{d}$ (with Observer 12 and Transform Block 11), this error is integrated in the integrator (Module 13), whose output value is the correction angle Δγ, whose value forces the rotation of the input voltage $u_{\alpha -ref}$$u_{\beta -ref}$, creating a corrected reference voltage $u_{\alpha -ref2}$$u_{\beta -ref2}$ for the inverter (Module 6). The system uses three correction loops. First, the current $i_{d}$ correction loop, which is described above, adjusts the position (using the *rotator*) of the voltage vector PMSM so as to minimize the error value of current $i_{d}$, while the reference value is maintained at a value of zero in accordance with the control strategy used in the reference drive model. The second loop estimates a load torque using Module 15. The proportional-integral structure (Module 15) based on the reference current $i_{q-ref}$ (output of Module 2) and estimated current ${\hat{i}}_{q}$ (using Modules 12 and 11) estimates the value of the load ${\hat{T}}_{l}$. The third loop modifies the speed setpoint for Module 2, using Modules 3 and 1. In the case of higher values of gain *k*, an additional block, the rate limiter (Module 17), may be used, which decreases the slope of the correcting speed value and increases the tolerance range for the motor parameter estimation error. Correction of the reference speed is necessary in a situation whereby the action of the rotator and the rotation speed of the voltage vector $u_{ref2}$ are different than the speed of rotation of the voltage vector $u_{ref}$. As a result of that correction, if a speed error occurs, the reference model follows the operating point of the real object. Hence, the system operates differently from the typical MFC, where if a difference in object output value and model output occurs, the supplementary controller acts on the object.

## 5. Results

**Figure 4:**
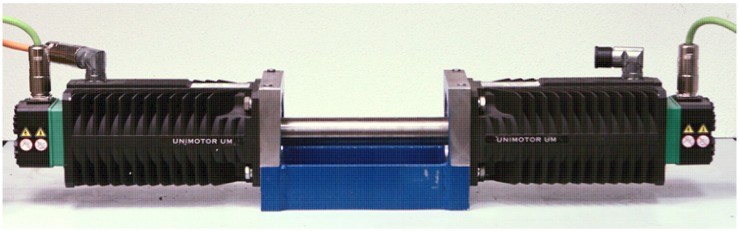
Mechanical setup: twin PMSM with a stiff shaft.

In order to verify experimentally the proposed estimation methods, a laboratory stand has been designed and built. It consists of two similar PMSMs: the first is powered by the laboratory, three phase power IGBT inverter, controlled by the digital signal processor (DSP). The second, which acts as a controlled load, is powered by an industrial inverter. Both motors are coupled by a stiff shaft. The voltages and currents are measured using 12-bit A/D converters. The shaft position is measured using a precision incremental encoder. The general view of the mechanical setup (motors used in experiments) is shown in Figure 4.

All control algorithms were implemented in DSP by AnalogDevices (SHARC 21369) using the *C* language. For the calculation period, both the speed and current control loops were equal to 50 μs in the case where the estimation method was based on the Kalman filter. They were synchronized with a 10-bit PWM generator. In the case of the low-speed method, which was based on the reference model, the calculation period was equal to 100 μs. The motor shaft position was measured by an incremental encoder, and then, the raw signals were processed using FPGA and sent to DSP by parallel memory fields. The PWM symmetrical generator operated with a carrier frequency equal to 20 kHz or 10 kHz, dependent on the used sensorless control structure, and can be updated twice on pulse. The parameters of the current controller were determined according to the modulus optimum [1] using the IPstructure. The speed controller was prepared using the PI structure. The results presented in Section 5.1 were obtained in a typical structure control system using the Kalman filter, the control system shown in Figure 1. The results presented in Section 5.2 include tests of a control system containing a reference model, the structure of which is shown in Figure 2.

### 5.1. Unscented Kalman Filter

The operation of the control system with *unscented Kalman filter* for laboratory verification on the referenced setup from Figure 4 is presented below. There are two important references: first, changing the desired speed in a wide range and, second, load torque acting during constant speed operation.

#### 5.1.1. Speed Control

In order to test the behavior of observers under varying conditions of work, it was decided to use tests involving a change of reference speed $\omega_{r}^{*}$. The first investigation was performed as a speed reference in stages: start, constant speed region, reverse, and braking. The maximum modulus of reference speed in this case was $\frac{1}{3}$ of maximum speed: $1000\;\frac{\mathrm{rev}}{\min}=104.72\;\frac{\mathrm{rad}}{s}$.

**Figure 5:**
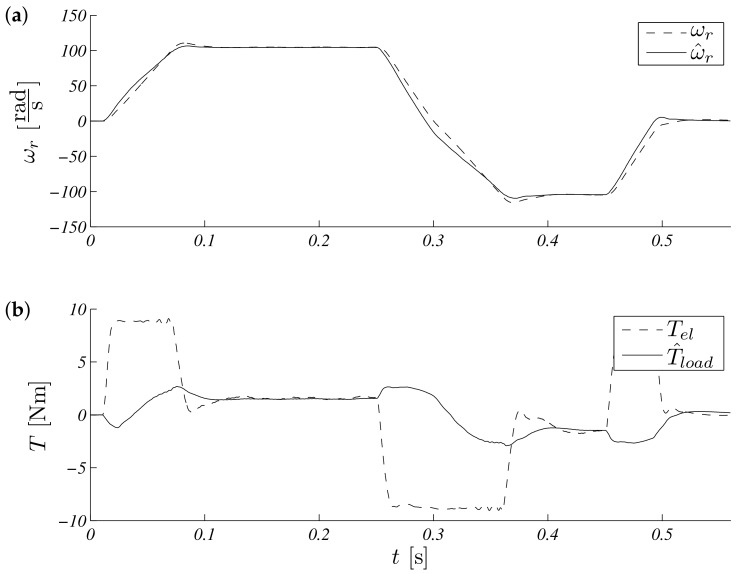
Unscented Kalman filter speed (**a**) and torque (**b**) response for reference speed $\omega_{r}^{*}=104.72\;\frac{\mathrm{rad}}{s}$.

**Figure 6:**
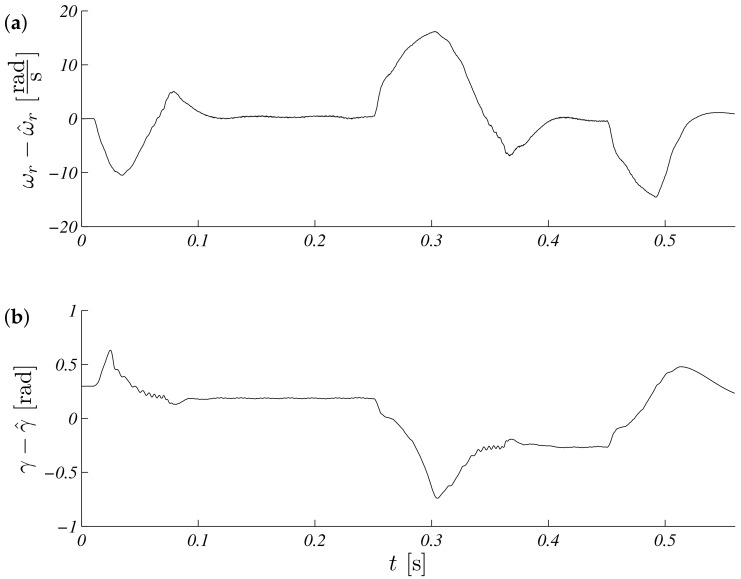
Unscented Kalman filter estimation speed (**a**) and torque (**b**) errors for referenced speed $\omega_{r}^{*}=104.72\;\frac{\mathrm{rad}}{s}$.

**Figure 7:**
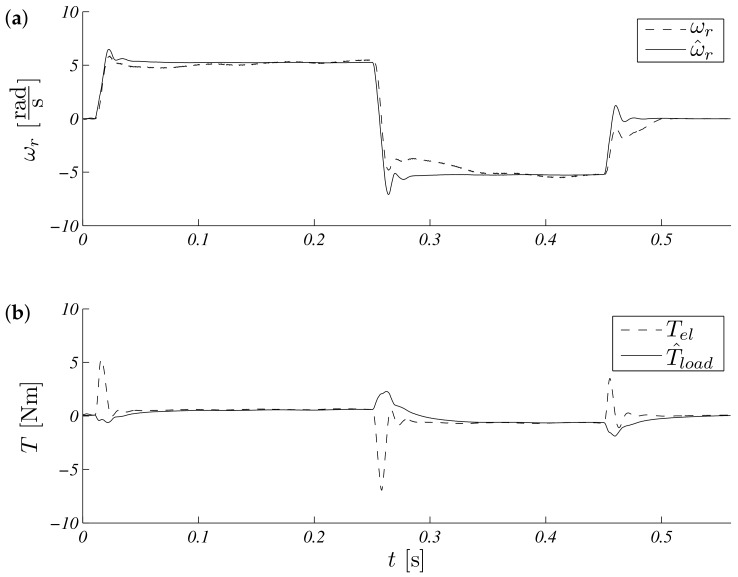
Unscented Kalman filter response speed (**a**) and torque (**b**) for referenced speed $\omega_{r}^{*}=5.28\;\frac{\mathrm{rad}}{s}$.

**Figure 8:**
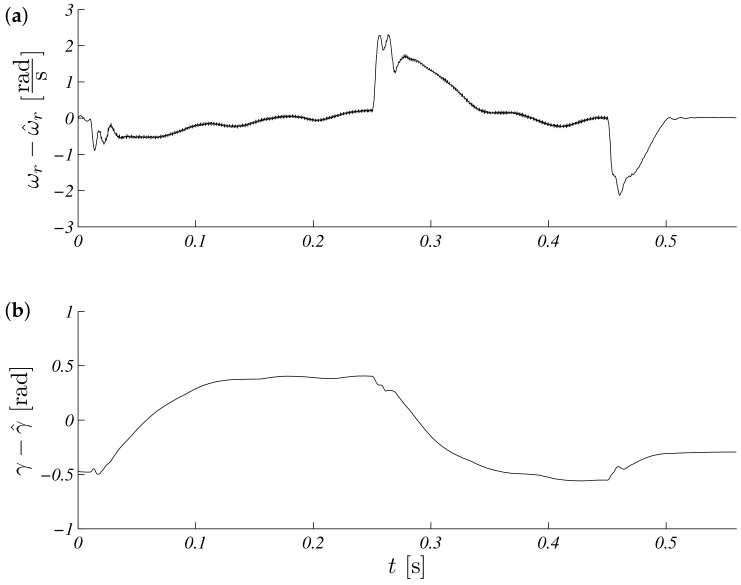
Unscented Kalman filter estimation speed (**a**) and torque (**b**) errors for reference speed $\omega_{r}^{*}=5.28\;\frac{\mathrm{rad}}{s}$.

The operation for this speed range is shown in Figure 5. Estimated speed overlap measured steady states, and a small difference is visible in transient states (Figure 5a). The process of estimating the load torque is presented in Figure 5b. It can be seen that, at a constant speed, the estimated load torque ceased to change its value. The error waveforms for the above test are shown in Figure 6. It is visible that the most critical situation included a zero speed crossing (in time just above 0.3 s and above 0.5 s, braking). An interesting point of the works control system with the observer is the work during real speeds near zero. The same shape as above is presented in Figure 7, but the maximum modulus is $\omega_{r}^{*}=5.28\;\frac{\mathrm{rad}}{s}$. Errors obtained during the experiment are presented in: Figure 8.

#### 5.1.2. Load Torque

**Figure 9:**
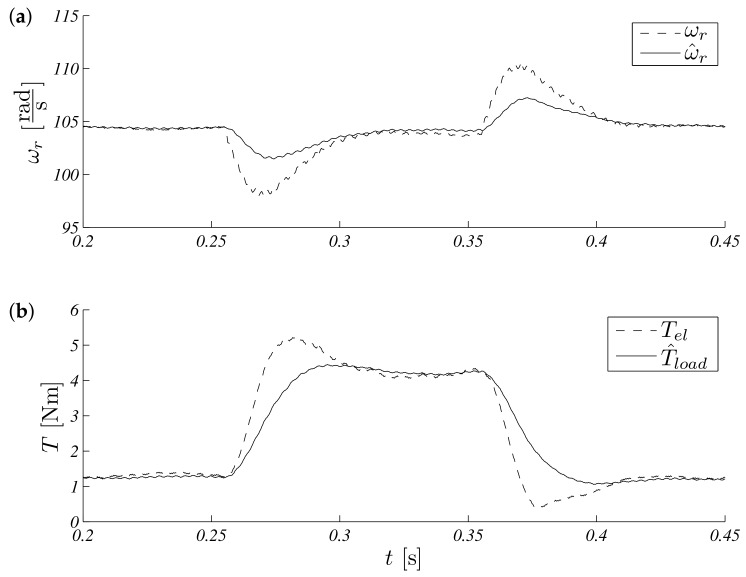
Unscented Kalman filter speed (**a**) and torque (**b**) response for load torque $T_{l}=3\;\mathrm{Nm}$.

**Figure 10:**
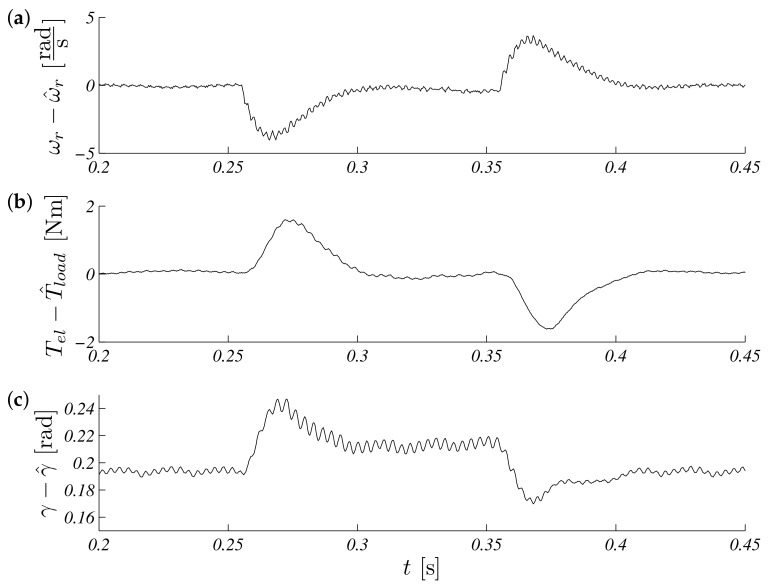
Unscented Kalman filter estimation speed (**a**), torque (**b**) and position (**c**) errors for load torque $T_{l}=3\;\mathrm{Nm}$.

The presented formal approach of reference signals consisted of a few important stages of operation like: zero speed with no initial (random) observer values, demand speed, positive and negative steps, and external load torque step. The constant reference speed was equal to $104.72\;\frac{\mathrm{rad}}{s}$ and disturbance; additional load torque was equal 3Nm. The collected results are presented in Figure 9 with associated errors in Figure 10.

### 5.2. Low-Speed Structure Using the Reference Model

Preliminary simulation studies were designed to determine the robustness of the analyzed control structure on the inaccurate estimation of the parameters of the power circuit of the drive. The presented selected waveforms showed the result of the following experiment: start-up to the speed of $4\frac{\mathrm{rad}}{s}$; at time 0.1s, the motor was loaded, and at time 0.25s, the load $T_{l}$ was turned off. All tests were performed in a closed system, where the control chain used the position information, which was estimated by the observer. The modeled system was powered directly, without considering the step-down transformer. The *real* motor inverter was simplified to zero order hold, and the inverter in the reference module was omitted (as in the reference model implemented in the real system).

#### 5.2.1. Simulations Research

**Figure 11:**
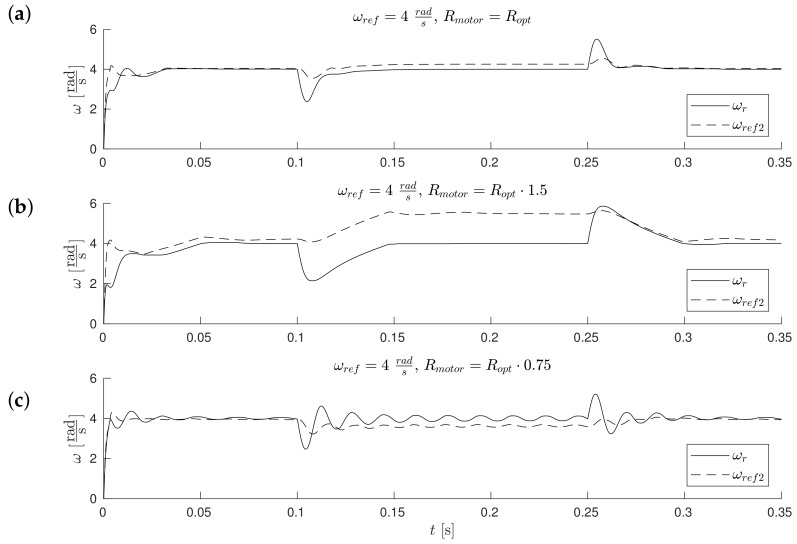
Measured $\omega_{r}$ and corrected reference $\omega_{ref2}$ speeds waveforms for different estimation errors of motor resistance (the initial position equals $30^{\circ }$) (**a**) $R_{motor}=R_{opt},$ (**b**) $R_{motor}=1.5\cdot R_{opt},$ (**c**) $R_{motor}=0.75\cdot R_{opt}$.

**Figure 12:**
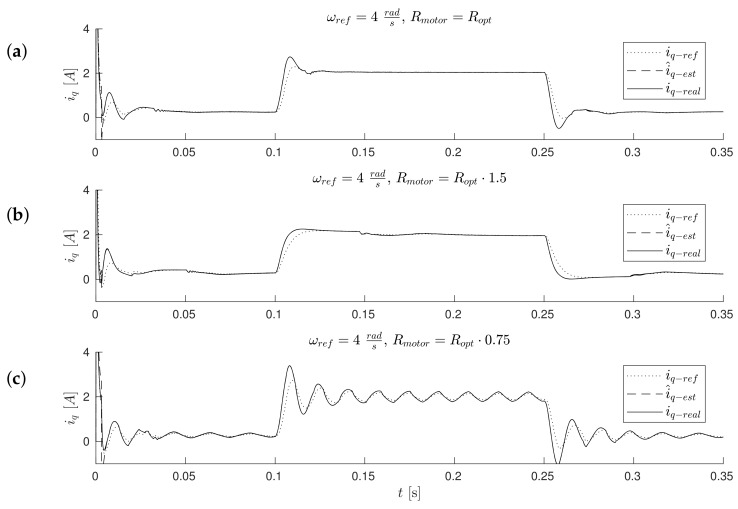
Waveforms of reference (model) $i_{q-ref}$, estimated ${\hat{i}}_{q-est}$, and measured $i_{q-real}$ currents for different estimation errors of motor resistance (the initial position equals $30^{\circ }$) (**a**) $R_{motor}=R_{opt}$, (**b**) $R_{motor}=1.5\cdot R_{opt},\;$(**c**) $R_{motor}=0.75\cdot R_{opt}$.

**Figure 13:**
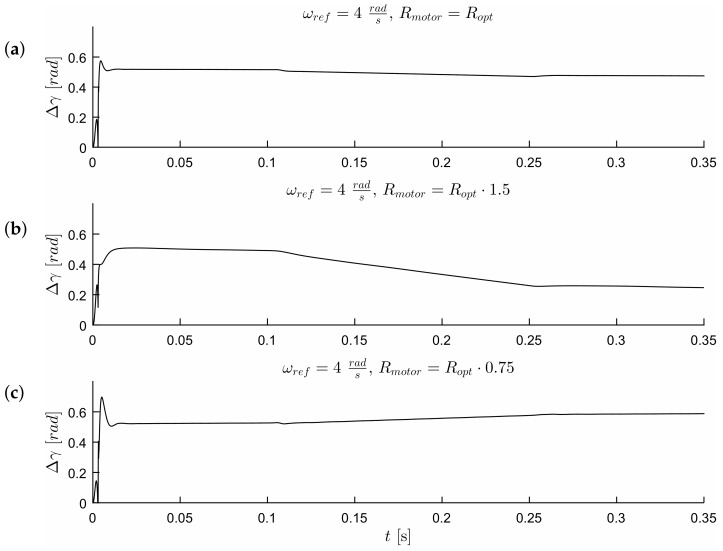
Waveforms of correction angle Δγ for different estimation errors of motor resistance (the initial position equals $30^{\circ }$) (**a**) $R_{motor}=R_{opt}$, (**b**) $R_{motor}=1.5\cdot R_{opt},$ (**c**) $R_{motor}=0.75\cdot R_{opt}$.

The waveforms of Figure 11, Figure 12, Figure 13 and Figure 17 were obtained for gain *k* of the correction angle Δγ (Figure 2, Module 3) with the optimal value in terms of the dynamics of tracking the speed setpoint. In Figures 15 and 16, gain *k* had twice reduced its value. In this way, the robustness of the drive was increased in the case of incorrect estimation of the parameters of the motor. Waveforms of Figure 11, Figure 12 and Figure 13 show the essential waveforms for different values of resistance of the motor (altered only in the machine), for an initial shaft position equal to $30^{\circ }$ (electric angle).

**Figure 14:**
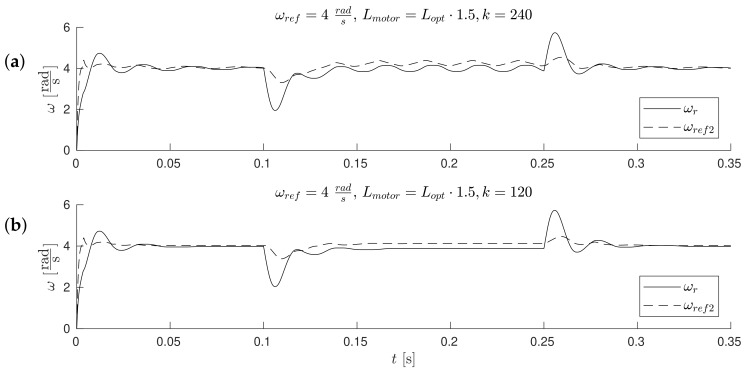
Waveforms measured $\omega_{r}$ and corrected reference $\omega_{ref2}$ speed waveforms, for the wrong estimation of motor inductance and for different values of gain *k* (amplification of correction angle input); the initial position equals $0^{\circ }$. (**a**) $L_{motor}=L_{opt}\cdot 1.5,\;k=240$, (**b**) $L_{motor}=L_{opt}\cdot 1.5,\;k=120$.

**Figure 15:**
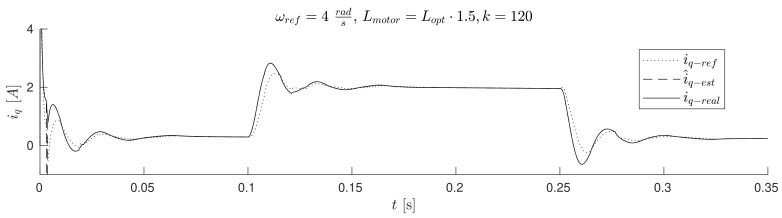
Reference $i_{q-ref}$, estimated ${\hat{i}}_{q-est}$, and measured $i_{q-real}$ current waveforms; wrong estimation of the inductance of the motor, reduced the value of gain *k*; the initial position equals $0^{\circ }$.

**Figure 16:**
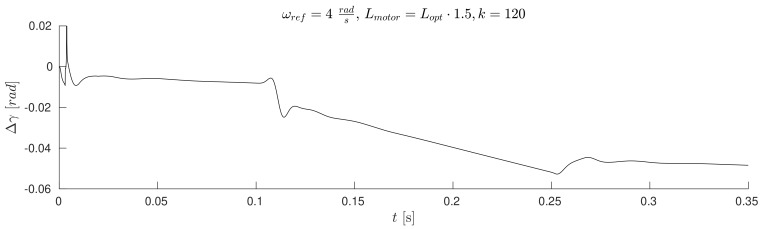
Correction angle Δγ; wrong estimation of the inductance of motor reduced the value of gain *k*; the initial position equals $0^{\circ }$.

**Figure 17:**
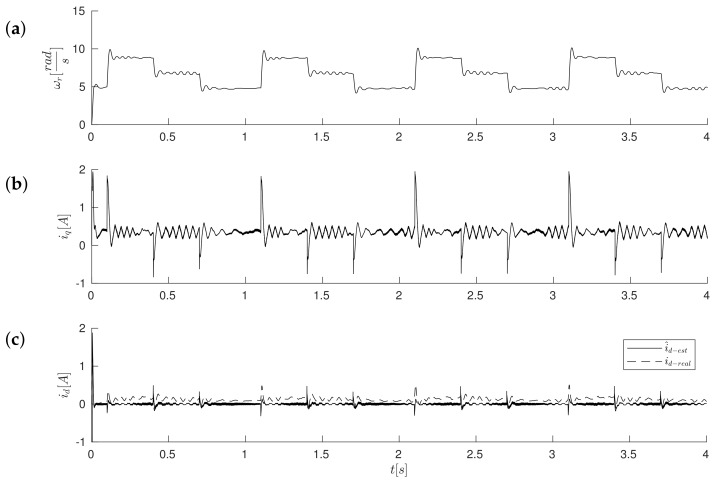
Simulation of low-speed control structure operation during speed changes $\omega_{ref}=8.8\to 6.8\to 4.8\;\left[\frac{\mathrm{rad}}{s}\right]$, corresponding to the experiment shown in Figure 18. Values of motor speed $\omega_{r}$ (**a**), currents $i_{q}$ (**b**) and $i_{d}$ (**c**) respectively.

**Figure 18:**
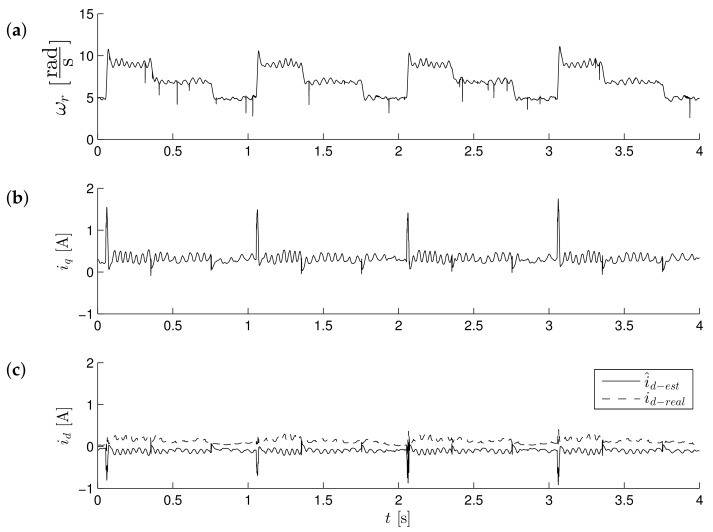
Experimental behavior of low-speed control structure operation during speed changes $\omega_{ref}=8.8\to 6.8\to 4.8\;\left[\frac{\mathrm{rad}}{s}\right]$. Values of motor speed $\omega_{r}$ (**a**), currents $i_{q}$ (**b**) and $i_{d}$ (**c**) respectively.

Figure 11a, Figure 12a and Figure 13a show the reference results for correctly-determined resistance (the optimum value is $R_{opt}$). Where the resistance value of the machine was increased by 50% of the value used in the control loop, the stability did not deteriorate significantly. In the case of a lower value by 25%, this clearly increased the oscillations. The figures show the waveforms of measured speed $\omega_{r}$ and adjusted reference speed $\omega_{ref2}$. It is noticed that the speed was adjusted, depending on the accuracy of the estimate, to a degree greater to or lesser than the set value. The compensation value was associated with the load of the machine. The waveforms in Figure 12 show the high correspondence of the current in the q-axis reference model ($i_{q-ref}$) and the measured current $i_{q-real}$ of the machine. The operation of the rotator is shown in Figure 13. Since the initial position of the shaft for this test was $30^{\circ }$ (0.52rad), after starting the drive, the correction angle value Δγ quickly tended to approach this value and then changed towards minimizing the d-axis component of the the current vector. The rate of change Δγ was related to the accuracy of estimation of the parameters and the value of the load of the machine. Figure 14 shows the effect of changing the factor of correction angle Δγ on the stability of the sensorless operation: even in the case of a large error in the estimation of inductance, which caused an oscillating course of the speed (Figure 14a), one can get a stable, non-oscillating operation of the drive. The result of this, however, was the increase in the steady state error of speed (Figure 14b). The speed waveforms shown in Figure 14b correspond to the current waveforms of Figure 15 and the waveform of angle correction Δγ of Figure 16. Finally, in Figure 17, the test sequence of reference speed response is shown, which was verified in laboratory conditions (Figure 18). In this case, the PMSM model was supplemented with additional non-linearity: in the function of the shaft position, a back EMF was modified in a sinusoidal manner. It is visible that in both cases (simulation and experiment), the waveforms were similar, especially in the case of (measured) speed waveforms (Figure 17a and Figure 18a). Only the differences in the waveform shape of the current $i_{q}$ (Figure 17b and Figure 18b) are visible in the moments of decreasing the reference speed (the minimum value of the current). Furthermore, the general characteristics of the currents’ shapes of $i_{d}$ were quite similar (Figure 17c and Figure 18c).

#### 5.2.2. Experimental Research

**Figure 19:**
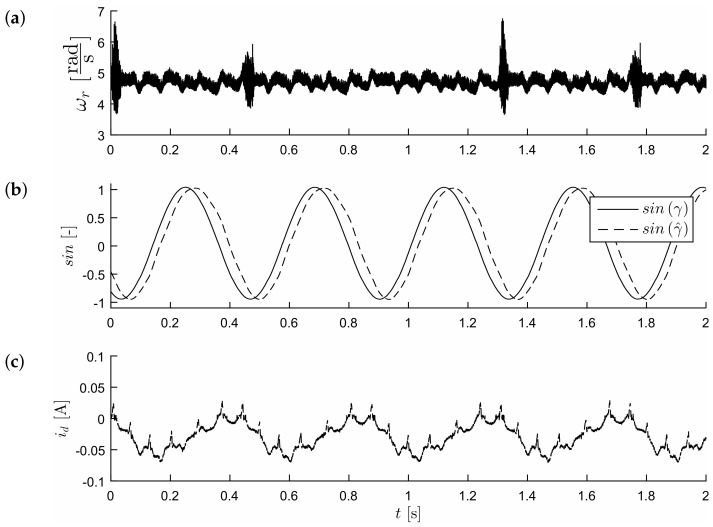
Low-speed control structure at steady state, $\omega_{ref}=4.8\frac{\mathrm{rad}}{s}$. Values of motor speed $\omega_{r}$ (**a**), position sine—sin(γ) (**b**) and *d* axis current $i_{d}$ (**c**) respectively.

Figure 18 and Figure 19 show experimental results for the control method with the reference model. The system operates as fully sensorless; a position sensor was used only to evaluate the accuracy of the estimation of the position of the shaft in the test control system. The first test compared the behavior of the model and the real drive. It consisted of the sequence of step changes in the speed command in the range $\omega_{ref}=8.8\to 6.8\to 4.8\;\left[\frac{\mathrm{rad}}{s}\right]$ (Figure 18). It proved the correct operation of the drive in transient states. There were visible pulses of estimated current ${\hat{i}}_{d-est}$ in times of reference speed changes; however, the real value of current $i_{d-real}$ was maintained near the referenced value equal to zero. Figure 19 shows the waveforms obtained in the steady state, for a speed of $4.8\frac{\mathrm{rad}}{s}$. One may notice the effects of periodic electromagnetic interference in the measured speed waveforms (Figure 19a) which, however, did not affect the reliability of the sensorless drive operation. One can notice that the value of the position (sine of position angle; this observer did not estimate the position angle) had a smooth path (also in the presence of external interference, it was visible in the course of the measured velocity), although the error was not close to zero (Figure 19b). The error in estimating the position was compensated by the action of the rotator, so that the measured current in the d-axis was maintained with reasonable accuracy at the (zero) value (Figure 19c). In the case of a bigger error value of a position estimation (as is visible in Figure 19), it was necessary to improve the accuracy of the current $i_{d}$ tracking by an additional compensation value $\Delta \gamma_{2}$, which in general is the function of the load. This additional value $\Delta \gamma_{2}$ was added to the output of the current $i_{d}$ corrector (the control input of the rotator was given the value $\Delta \gamma +\Delta \gamma_{2}$).

## 6. Conclusions

The paper showed the effects of the operation of the sensorless drive, using different control structures. Both structures were compared in a range of low speeds. It was shown that the system based on back EMF estimation can work properly in this speed range. All the results were obtained using sensorless operation with a closed-loop mode. This means that the sine and cosine of the motor shaft position used in the control were produced by the observers. Two control structures were used in the study. The first was a typical control structure with a UKF observer. The second was a new control structure with the reference model. The position observer, which was based on the back EMF estimation, turned out to be useful also at a low-speed range. In the case of the studied PMSM laboratory set, the lowest speed that gave stable operation was about $4-5\;[\frac{\mathrm{rad}}{s}]$.

The UKF observer of the mechanical quantities worked in every range of operation speed. Such a system provides an interesting research object and may find its use in industrial applications. The advantage of the sensorless structure with the Kalman filter is the possibility to estimate the speed and also its sign correctly, even in the case of speed reversal. The wide development of an experimental sensorless drive system is under way.

The presented new control structure, which used the drive model, was particularly effective in the field of low speed. The module called the rotator (Figure 2, Module 4) allowed compensating for the position error estimated by the observer. This allowed maintaining the zero (reference and real) value of the current in the *d*-axis with good accuracy, also in transient states. The main advantage of the sensorless structure with the reference model was that it reduced the need to estimate the speed, despite the fact that it was a speed control system. This is an important feature because it eliminated problems with speed estimation without the excitation of oscillations. The correct operation of the new control structure was checked by comparing the simulation with the experiment.

It is worth noting that both the presented estimation and the control schemes detected load torque. For the Kalman filter, this essential process was realized by including the load torque in the estimated state vector, and for the structure with the reference model, the additional PI unit was sufficient. These concepts were fully proven in different ways, which showed the good properties of the presented methods. In the current state of research, it seems that the new control structure with the reference model has reached a more advanced stage, because it provides more accurate control of *d*-axis and *q*-axis currents and also provides satisfactory accuracy of speed control.

## Appendix A

*Motor parameters:* Basic parameters of the PMSM are presented in Table A1 below.

**Table A1:** Parameters of the PMSM.

Parameter	Units	Value
*P*	kW	1.23
$T_{N}$	Nm	3.9
$R_{s}$	Ω	1.15
$L_{d}$	mH	6.8
$L_{q}$	mH	6.8
$\Psi_{m}$	Wb	0.254
